# Pharmacometabolomics Profiling of Preterm Infants Validates Patterns of Metabolism Associated With Response to Dexamethasone Treatment for Bronchopulmonary Dysplasia

**DOI:** 10.3389/fped.2022.898806

**Published:** 2022-06-10

**Authors:** Bradley Stockard, Cheri Gauldin, William Truog, Tamorah Lewis

**Affiliations:** ^1^Division of Clinical Pharmacology, Toxicology and Therapeutic Innovation, Department of Pediatrics, Children’s Mercy Hospital, University of Missouri–Kansas City School of Medicine, Kansas City, MO, United States; ^2^Division of Neonatology, Department of Pediatrics, Children’s Mercy Hospital, University of Missouri–Kansas City School of Medicine, Kansas City, MO, United States

**Keywords:** pharmacometabolomics, neonatology, pulmonology, dexamethasone, pharmacology

## Abstract

Bronchopulmonary dysplasia (BPD) is one of the most common health complications of premature birth. Corticosteroids are commonly used for treatment of BPD, but their use is challenging due to variability in treatment response. Previous pharmacometabolomics study has established patterns of metabolite levels with response to dexamethasone. We obtained additional patient samples for metabolomics analysis to find associations between the metabolome and dexamethasone response in a validation cohort. A total of 14 infants provided 15 plasma and 12 urine samples. The measure of treatment response was the calculated change in respiratory severity score (deltaRSS) from pre-to-post treatment. Each metabolite was assessed with paired analysis of pre and post-treatment samples using Wilcoxon signed rank test. Correlation analysis was conducted between deltaRSS and pre-to-post change in metabolite level. Paired association analysis identified 20 plasma and 26 urine metabolites with significant level difference comparing pre to post treatment samples (*p* < 0.05). 4 plasma and 4 urine metabolites were also significant in the original study. Pre-to-post treatment change in metabolite analysis identified 4 plasma and 8 urine metabolites significantly associated with deltaRSS (*p* < 0.05). Change in urine citrulline levels showed a similar correlation pattern with deltaRSS in the first study, with increasing level associated with improved drug response. These results help validate the first major findings from pharmacometabolomics of BPD including key metabolites within the urea cycle and trans-4-hydroxyproline as a potential marker for lung injury. Ultimately, this study furthers our understanding of the mechanisms of steroid response in BPD patients and helps to design future targeted metabolomics studies in this patient population.

## Introduction

Bronchopulmonary dysplasia (BPD) is a lung disease occurring in preterm infants resulting from lung structure immaturity, ventilator-associated lung injury and inflammation. In the US, BPD is one of the most common complications of premature birth with approximately 18,000 infants impacted annually (1). This accounts for approximately 25% of infants with birth weight lower than 1,500 g (2–4). Despite the high rate of occurrence in the preterm infant population, there is no FDA approved treatment indicated for BPD. Corticosteroids are commonly used for prevention or management of BPD symptoms. Dexamethasone is a glucocorticoid that is used primarily for its anti-inflammatory and immunosuppressant effects. In cases of bronchopulmonary dysplasia, dexamethasone helps facilitate weaning from the ventilator. Common side effects of dexamethasone in pre-term infants can include hyperglycemia, hypertension, and GI bleeding/perforation. Unfortunately, there are a number of difficulties associated with corticosteroid use in preterm infants. This can include interpatient variability in treatment response and clinical effects. Compounding this variability in treatment response, there are no predictive factors that can help determine which patients will respond to treatment.

The growing field of pharmacometabolomics has emerged as a powerful method for furthering the understanding of mechanisms contributing to variability in drug response for many different fields of medicine. The patient metabolome can be measured at both pretreatment and post-treatment periods of care. Ideally, the metabolome measured pretreatment and a difference in the metabolome measured post-treatment, can help illustrate the metabolic mechanisms of variable drug response. These metabolome baseline measurements and changes could potentially serve as signatures for predicting drug response and be used clinically to help determine the likelihood of drug response prior to treatment or early in the treatment course. Furthermore, if these metabolome changes can be replicated in multiple studies, the evidence for a mechanistic relationship between metabolism and drug response is strengthened, and the potential for a predictive metabolic signature of drug response is further supported.

We previously conducted a pharmacometabolomics study on a pilot cohort of preterm infants less than 32 weeks gestation at birth who received dexamethasone treatment for BPD (5). Our results showed metabolites with a significant difference in abundance when comparing pretreatment to post-treatment metabolite levels. In order to validate the initial findings and confirm metabolites for future targeted analyses in larger cohorts, we obtained additional patient samples for pharmacometabolomics analysis. Our goal with this analysis is to conduct a replication cohort dexamethasone pharmacometabolomics study on preterm infant urine and plasma samples in order to identify and confirm metabolic changes associated with drug response.

## Materials and Methods

### Subjects and Study Design

Subject enrollment and study design followed the protocol described previously (5). To summarize, this replication cohort study was conducted under the approval by the Children’s Mercy Hospital institutional review board (IRB) prior to patient enrollment. Parental consent was obtained in accordance with IRB regulations. Starting in October 2016, all preterm infants less than 32 weeks gestation at birth and treated with systemic dexamethasone per clinical care were eligible for enrollment. Essentially all infants were requiring substantial ventilatory support, either intubated or via non-invasive assisted ventilation. Exclusion criteria included any patients with an active infection (the DART (Dexamethasone: A Randomized Trial) course was deferred until infection was resolved), coexisting significant structural heart disease, non-prematurity related cause of their pulmonary disease, complex structural, or known genomic differences, and congenital difference in lung or airways development. Patient demographics and clinical data were obtained from the clinical chart and by speaking with bedside clinicians. For this analysis, we used data from the first course of systemic dexamethasone for each child, as well as data from the second course of dexamethasone from two patients.

The DART course was used at our institution to standardize dexamethasone dosing for BPD. The course consisted of 0.15 mg/kg/daily for 3 days, then 0.1 mg/kg/day for 3 days, then 0.05 mg/kg/day for 2 days, then 0.02 mg/kg/day for 2 days. The total dose was 0.89 mg/kg. Clinical outcome was measured as short-term phenotypic response to dexamethasone. The Respiratory Severity Score (RSS = mean airway pressures x FiO2) was calculated prior to treatment (baseline) and on day 7 of treatment (drug response). In order to account for intraindividual variability, the average RSS for a 24-h period was collected. RSS was calculated as the mean airway pressure × fraction of inspired oxygen (FiO2) (ranging from 21 to 100%).

Blood and urine samples were collected two times, once in the 24 h prior to starting systemic dexamethasone (pre-treatment) and again at day 3–6 after starting systemic dexamethasone (post-treatment). Post-treatment blood and urine sample collection timing was conducted during the steroid course and determined by and paired with scheduled blood draws for clinical labs within the target window. Samples were collected in the NICU and then either briefly refrigerated or immediately processed. Urine and plasma samples were aliquoted and stored at –80^°^C until metabolomic assay.

### Untargeted Metabolic Assessment

Methods for metabolomics analysis were described previously (5). Briefly, plasma and urine samples were submitted for untargeted metabolomics analysis to the West Coast Metabolomics Center at UC Davis. Untargeted metabolomics profiling was performed using an Agilent 7890A gas chromatograph and a Leco Pegasus IV time-of-flight mass spectrometer. BinBase database was used to identify metabolites by retention index, align mass spectra, and perform gap filling. Data was reported as mass spectral peak height.

### Statistical Analysis

Peak height values were subjected to data preprocessing prior to analysis. Missing value estimation was performed by removing metabolites with more than 30% of values missing among the patient samples. Any remaining missing values were replaced with a calculated small value (half of the minimum positive value for that metabolite reported in the raw data). Further data processing also included normalization by sum peak intensity of all known metabolites as well as log transformation. Categorical statistical analysis was performed on the MetaboAnalyst 5.0 platform (6). Patient pretreatment and post-treatment samples were paired for categorical analysis. The difference in metabolite abundance pre and post-treatment was tested using Wilcoxon signed rank test. Boxplots created from the data analysis featured quartiles 1 and 3 at the bottom and top of the box, respectively. Boxplot features included a horizontal line representing the median, an upper whisker of Q3 + 1.5*IQR and a lower whisker of Q1 – 1.5*IQR. Significance threshold was set at *p*-value < 0.05. Adjustment for multiple comparisons was done using Benjamini and Hochberg’s false discovery rate method. Statistical analysis on change in metabolite level and RSS values was performed using Pearson correlation analysis. Correlation analysis was conducted to determine if changes in metabolite abundance from pretreatment to post-treatment was associated with change in RSS, representing clinical response to dexamethasone therapy. Significance threshold was set at *p*-value < 0.05.

## Results

**FIGURE 1:**
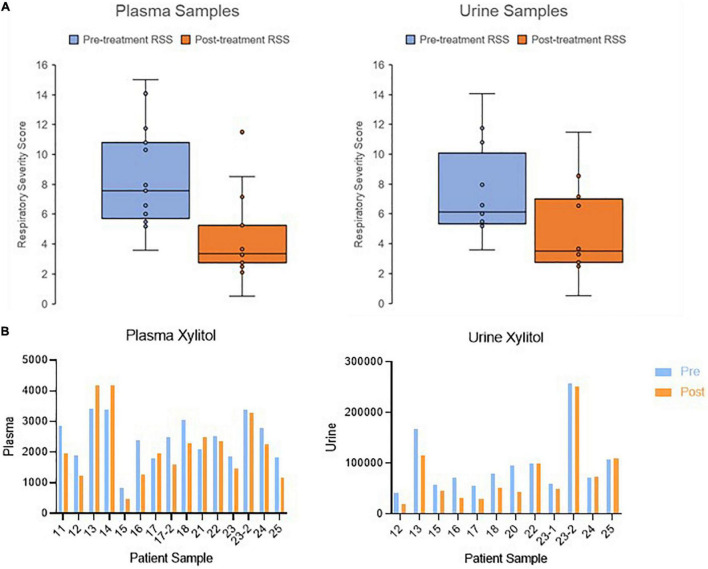
Change in clinical outcome and metabolite abundance from pretreatment to post-treatment. **(A)** Boxplots showing the change in respiratory severity score in patients with collected plasma and urine samples. **(B)** Bar graph showing the change in xylitol as an example metabolite. Metabolite abundance is displayed with arbitrary units of measurement.

In this replication cohort, 14 infants provided 15 matched pretreatment and post-treatment plasma samples and 12 matched pretreatment and post-treatment urine samples. Two patients, 17 and 23, provided multiple samples from both first and second rounds of treatment with dexamethasone. Detailed demographic information for the patient cohort is provided in Table 1. Comparison of pretreatment and post-treatment RSS values for plasma and urine samples are shown in Figure 1.

**TABLE 1:** Demographics and respiratory data.

Discovery cohort data									
**Patient #**	**GA (weeks)**	**BW (g)**	**Race**	**DOL steroid** **started**	**Pre-RSS**	**Post-RSS**	**deltaRSS**	**Plasma** **sample**	**Urine** **sample**
Median	25 0/7	663	–	36	6.48	2.6	–2.3	–	–
IQR	24 2/7-26 0/7	465–790	–	27–115	4.29–9.02	1.86–3.21	–5.32 to –1.79	–	–

**Replication cohort data**									
**Patient #**	**GA (weeks)**	**BW (g)**	**Race**	**DOL steroid** **started**	**Pre-RSS**	**Post-RSS**	**deltaRSS**	**Plasma** **sample**	**Urine sample**

11	25 3/7	704	AA	17	10.32	2.84	–7.48	X	–
12	24 3/7	1,000	WH	18	6.02	3.66	–2.36	X	X
13	29 3/7	1,330	WH	27	10.8	11.5	0.7	X	X
14	24 1/7	510	WH	49	15	3.7	–11.3	X	U
15	25 3/7	840	AA	158	5.7	3.37	–2.33	X	X
16	25 3/7	510	AA	40	14.08	3.69	–11.11	X	X
17	25 0/7	820	O	29	11.76	2.76	–9	X	X
17-2	25 0/7	820	O	61	7.7	2.1	–5.6	X	U
18	25 2/7	879	WH	25	5.17	2.5	–2.67	X	X
20	23 1/7	580	AA	15	5.28	6.54	1.26	–	X
21	24 6/7	660	WH	24	7.56	5.27	–2.29	X	–
22	24 6/7	970	WH	41	6.6	2.8	–3.8	X	X
23	24 2/7	765	WH	30	6.24	7.16	0.92	X	X
23-2	24 2/7	765	WH	104	5.48	8.54	3.06	X	X
24	24 1/7	680	AA	29	7.95	3.29	–4.66	X	X
25	26 2/7	1,090	WH	88	3.57	0.5 (NC)	–3.07	X	X
*T-test									
*p*-value	0.7027	0.3512	–	0.3441	0.2572	0.1580	0.9837	–	–

*AA, African American; BW, birthweight; DOL steroid, day of life (age) when infant started steroids; GA, gestational age at birth; IQR, interquartile range; NC, Nasal Canula; O, Other; RSS, Respiratory Severity Score; U, Post treatment samples unavailable; WH, white. Negative deltaRSS indicates improved lung function.*

*T-test comparing demographics and baseline data between discovery and validation cohorts.

A total of 690 metabolites were measured in plasma and urine samples. Within this metabolite set, 135 metabolites were identified in plasma samples and 178 were identified in urine samples. Categorical analysis of pretreatment vs. post-treatment plasma samples identified 18 metabolites with statistically significant difference in abundance comparing pre and post-treatment. Among these significant metabolites, three were replicated from the same analysis in the pilot cohort. These metabolites are xylitol, trans-4-hydroxyproline, and p-hydroxyphenyllactic acid. One significant metabolite, gluconic acid lactone, was significant in the free acid form in the serum analysis of the previous study, but also significant in the lactone form in the urine sample regression analyses of deltaRSS.

**FIGURE 2:**
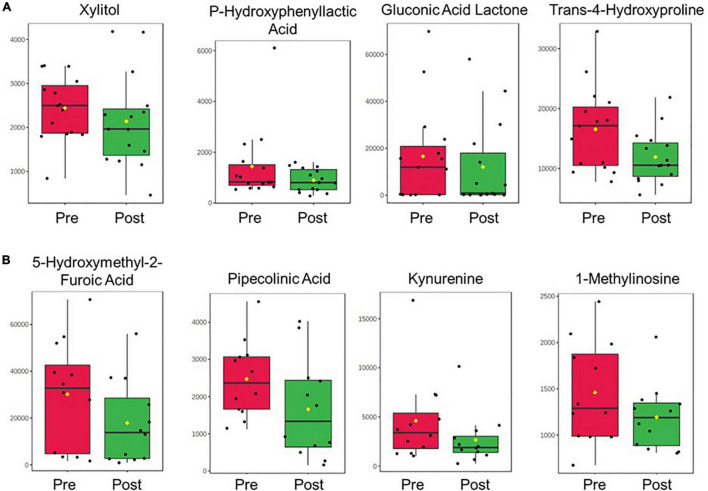
Boxplots displaying the pre-to-post-treatment difference in abundance for the replicated metabolites in **(A)** plasma and **(B)** urine between the discovery and replication cohort studies. Metabolite abundance is displayed with arbitrary units of measurement. Yellow dots represent the group mean.

In urine samples, 26 metabolites were found to be statistically significantly different between pretreatment and post-treatment samples. Among these significant metabolites, four were replicated with the results of the same analysis conducted in the discovery cohort study. These metabolites are 5-hydroxymethyl-2-furoic acid, pipecolinic acid, kynurenine, and 1-methylinosine. The significant results from both plasma and urine sample analysis are included in Table 2. The pre to post treatment comparison of replicated metabolite abundances are shown in Figure 2.

**TABLE 2:** List of metabolites significantly associated with pre-post dexamethasone treatment comparison.

Metabolites	Fold change	Direction	*P-value* ^a^
***Plasma***			
**Xylitol**	0.73647	Down	0.0002
Kynurenine	0.62206	Down	0.0006
Hypoxanthine	4.0073	Up	0.0009
Urea	1.451	Up	0.0015
5-Hydroxymethyl-2-furoic acid	0.55663	Down	0.0043
Pseudo uridine	0.79005	Down	0.0054
**P-Hydroxylphenyllactic Acid**	0.64733	Down	0.0067
Gluconic acid lactone*	0.45612	Down	0.0103
Ribitol	0.81318	Down	0.0103
Uracil	0.86725	Down	0.0103
Xylulose	0.71414	Down	0.0103
Uric acid	1.2145	Up	0.0151
Uridine	1.3129	Up	0.0151
Xylose	0.85594	Down	0.0151
Ethanolamine	0.66029	Down	0.0181
Threonine	0.73213	Down	0.0181
Inosine	3.3745	Up	0.0215
Serine	0.8359	Down	0.0256
**Trans-4-hydroxyproline**	0.6678	Down	0.0256
Butane-2,3-Diol	1.7161	Up	0.0413
***Urine***			
P-hydroxylphenyllactic acid	0.46969	Down	0.0005
Oleic acid	1.4567	Up	0.0010
Xylitol	0.69276	Down	0.0010
Glycyl-proline	0.56812	Down	0.0010
Proline	0.34309	Down	0.0015
Palmitic acid	1.328	Up	0.0024
Aminomalonate	0.47598	Down	0.0024
Phthalic acid	0.66116	Down	0.0049
Creatinine	0.57385	Down	0.0049
Phenol	1.9054	Up	0.0068
Orotic acid	0.85041	Down	0.0068
3-Hydroxypropionic acid	0.68286	Down	0.0068
Threonine	0.39941	Down	0.0068
**5-Hydroxymethyl-2-furoic acid**	0.51825	Down	0.0093
Citric acid	0.64898	Down	0.0161
Stearic acid	1.3958	Up	0.0210
Glycine	0.55962	Down	0.0210
**Pipecolinic acid**	0.47561	Down	0.0210
Aspartic acid	0.47046	Down	0.0210
Methionine	0.70898	Down	0.0269
Thymine	0.68841	Down	0.0269
**Kynurenine**	0.5464	Down	0.0269
Threitol	1.1266	Up	0.0342
**1-Methylinosine**	0.82274	Down	0.0342
Phenylalanine	0.73404	Down	0.0342
Citramalic acid	1.2848	Up	0.0425

*Bold, Metabolite found significant in pre-post comparison for both discovery and replication cohorts.*

*^a^P-value included in the table is unadjusted for multiple comparisons.*

*Discovery cohort showed significant association with free gluconic acid form in serum and lactone form in urine.

**FIGURE 3:**
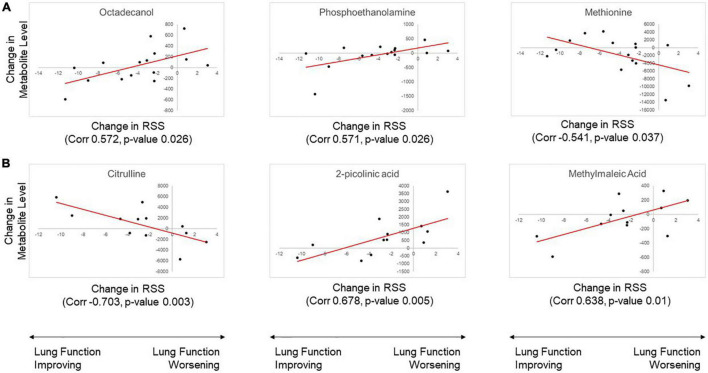
Results of correlation analysis for change in metabolite level with change in Respiratory Severity Score (RSS). The three metabolites with the most significant correlation in **(A)** plasma and **(B)** urine samples are shown. Metabolite abundance is displayed with arbitrary units of measurement.

Correlation analysis was conducted to determine if change in metabolite abundance was associated with change in RSS (clinical response). Analysis of plasma samples identified four metabolites with statistically significant association. Urine sample analysis identified eight metabolites with statistically significant association. Significant results from the analysis of plasma and urine samples are shown in Table 3. Among these significant metabolites, change in urine levels of citrulline showed a significant association with change in RSS score in both the original and replication cohort studies. Both association analyses also showed the same directional trend, with a positive change in RSS score (indicating poor response to therapy) associated with significantly lower levels of urine citrulline. Correlation plots of the top three most significant metabolites in plasma and urine samples are shown in Figure 3.

**TABLE 3:** Metabolites with change in abundance significantly associated with change in RSS.

Metabolites	Correlation coefficient	*p*-value
***Plasma***		
Octadecanol	0.572	0.026
Phosphoethanolamine	0.571	0.026
Methionine	–0.541	0.037
Maltotriose	0.519	0.048
***Urine***		
**Citrulline**	–0.703	0.003
2-Picolinic acid	0.678	0.005
Methylmaleic acid	0.638	0.010
Isoleucine	0.593	0.020
Sorbitol	–0.559	0.030
Indole-3-acetate	0.557	0.031
Aminomalonate	0.548	0.035
2,4-Hexadienedioic acid	0.540	0.038

*Bold, Metabolite found significant in correlation analysis for both discovery and replication cohorts.*

## Discussion

Adequate therapy for BPD prevention or treatment continues to be a major challenge in neonatology. Response to dexamethasone therapy, a common drug used clinically, shows high interpatient variability and is highly unpredictable. As we saw in our previous study, we again observe a wide range of clinical response within our replication cohort with a group of responders and non-responders. There are a variety of proposed mechanisms for the variable response to steroids in this patient population, and many of them involve metabolic activity and pathways. Pharmacometabolomics is an emerging powerful tool for effectively capturing the differences in metabolite levels between patients that can be linked to differences in treatment response. In our previous cohort study, we showed a number of metabolites and their relevant pathways that were significantly associated with treatment outcomes in BDP patients. In this replication cohort study, we are once again able to show a substantial number of metabolites associated with treatment response, with several of these metabolites showing overlap between the pilot and replication cohorts.

Among the replicated metabolomics results between our analyses of metabolite abundance affected by dexamethasone therapy, trans-4-hydroxyproline is one the most extensively discussed and relevant to BPD pathophysiology. In the prior study and this study, trans-4-hydroxyproline was found to be decreased in serum following dexamethasone treatment. Previous studies have identified trans-4-hydroxyproline as elevated in patients with idiopathic pulmonary fibrosis, suggesting that it could serve as an indicator for lung injury and fibrosis (7). Our replication cohort study showed a consistently significant association between trans-4-hydroxyproline levels and dexamethasone treatment. The metabolite showed a fold change of 0.6678 with a downward trend following treatment, providing further evidence of the relationship between trans-4-hydroxyproline and dexamethasone therapy. Since our first metabolomics cohort study, additional articles have been published on the subject of trans-4-hydroxyproline association with pulmonary fibrosis. There are significantly elevated levels of 4-hydroxyproline in exhaled breath analysis of idiopathic pulmonary fibrosis patients as compared to control (8). Also, serum 4-hydroxyproline, along with L-arginine, could serve as an effective diagnosis biomarker for the early phase of pulmonary fibrosis. This was further supported by showing that arctiin and arctigenin can downregulate serum 4-hydroxyprogresterone levels in cases of pulmonary fibrosis associated with silicosis (9). With further evidence from our replication cohort as well as additional published articles, trans-4-hydroxyproline continues to be a promising potential marker for lung fibrosis and injury, as well as drug response, in preterm infants at risk for BPD.

Gluconic acid lactone was significantly decreased in plasma samples following dexamethasone treatment. This replicates the results from the discovery cohort study, which showed a substantial decrease of the free form of gluconic acid following steroid treatment as well as a negative correlation with RSS score in urine samples. Gluconic acid metabolism continues to be poorly understood, and its role in BPD progression is unclear. However, gluconate concentrations can influence the hexose monophosphate shunt (10, 11). This shunt contributes to the production of ribose-5-phosphate, which leads to nucleotide synthesis through the pentose phosphate pathway. Interestingly, our results also showed an association of multiple metabolites in the pentose interconversion pathway with dexamethasone therapy. These include xylulose, ribitol, and xylitol, which were also significant in the discovery cohort study. The repeat significant association of these metabolites suggest an important role for the pentose phosphate pathway in response to dexamethasone therapy. This is supported by multiple studies that have proposed a connection between increased pentose phosphate pathway (PPP) activity, pulmonary hypertension, and BPD (12, 13). A recent study explored the relationship between BPD related hyperoxia and PPP activity. The authors found that hyperoxia caused increased PPP activity, which induced abnormal endothelial cell development in neonatal mice (14). Another recent study showed that glycolysis and PPP induction by interleukin-1 beta co-occurred with induction of pro-inflammatory signaling pathways in pulmonary tissues (15).

Our results show multiple metabolites involved in the urea cycle of arginine biosynthesis significantly associated with steroid therapy across both of our analyses. The pre-post dexamethasone treatment analysis showed a significant increase in the metabolite levels of urea in plasma samples and aspartic acid in urine samples. The change in RSS correlation analysis showed significant negative correlation of citrulline levels in urine samples with RSS. Results of our discovery cohort study also showed a significant association of citrulline levels with clinical response grouping of BPD patients, with lower levels of citrulline also associated with poorer response to therapy. Citrulline is produced through arginine catabolism as a byproduct of nitric oxide synthesis catalyzed by nitric oxide synthases. Citrulline can also serve as a precursor to arginine as the reactions are bidirectional. Several studies have proposed a link between citrulline and the urea cycle with BPD and pulmonary hypertension (PH) (16, 17). One study showed that citrulline plasma levels lower than 29 μmol/L were predictive of PH secondary to BPD (18). The authors of this study, Montgomery et al. ultimately propose that citrulline could potentially serve as a therapeutic target for PH secondary to BPD in preterm infants. Another study showed that a particular arginase-1 single nucleotide polymorphism (SNP), rs2781666, protects against the development of PH secondary to BPD by increasing synthesis of nitric oxide and reducing urea synthesis (19). In summary, results in both our discovery and replication cohort studies show that urea cycle metabolites, particularly citrulline, are closely related to BPD treatment response, and they could potentially serve as predictors of BPD development and response to dexamethasone therapy with further investigation.

Our replication study shares many of the strengths and weaknesses with our discovery cohort. Once again, we recruited a group of preterm infants with an even distribution of responders and non-responders, and we were able to pair pre and post treatment samples in most patients for analysis. NICU treatment protocol was the same between studies, so mg/kg dosing of dexamethasone was the same for both cohorts. An additional strength is inherent to the nature of replication studies in that we were able to support the findings of our discovery study by replicating several of the results with equivalent analyses. Lack of reproducibility is an ongoing crisis in many fields of medical research. By reproducing several of the significant findings of our discovery cohort study, we can begin to alleviate these concerns and greatly strengthen the confidence in our conclusions. Our study did again only involve untargeted metabolomics analysis, which provides relative metabolite abundance rather than absolute quantification. However, with a set of reproducible findings of metabolites and their pathways associated with dexamethasone response, we can approach the design and selection of targeted metabolomics panels with greater confidence. We are currently recruiting a large multi-site cohort for targeted metabolomics analysis, building on the strengthened findings from this study.

Metabolomics profiling continues to develop as a powerful tool for hypothesis generation and investigation of the biological mechanisms influencing response to pharmacotherapy. Since the publication of our previous study, several additional articles have been published on the relationship between metabolic activity with BPD and its comorbidities, as referenced previously. Metabolomics have been used to investigate several other disease states relevant to the neonatal patient population as well (20–24). Our findings contribute to this growing exploration of the link between metabolism and variable treatment response in neonates as well as strengthen several of the findings we have published previously. Ultimately, pharmacometabolomics profiling has the potential to discover powerful biomarkers than can be collected during patient care in order to predict risk of disease development and response to established pharmacotherapy for the individual patient. Furthermore, identification of relevant metabolites and their pathways can present new targets for the development of novel therapies for this patient population. The results of our study continue to expand on the investigation of pharmacometabolomics in preterm infants and the strength of reproducible results allow us to continue to narrow our focus on the relevant metabolic pathways for improving treatment outcomes in preterm infants with BPD.

## Data Availability Statement

The raw data supporting the conclusions of this article will be made available by the authors, without undue reservation.

## Ethics Statement

The studies involving human participants were reviewed and approved by the Children’s Mercy Hospital Institutional Review Board. We obtained written informed consent from the parents.

## Author Contributions

BS, CG, WT, and TL wrote the manuscript. TL and WT designed the research. TL, CG, and WT performed the research. TL and BS processed metabolomics data and performed statistical analysis. All authors contributed to the article and approved the submitted version.

## Conflict of Interest

The authors declare that the research was conducted in the absence of any commercial or financial relationships that could be construed as a potential conflict of interest. The handling editor declared a past co-authorship with one of the authors, TL.

## Publisher’s Note

All claims expressed in this article are solely those of the authors and do not necessarily represent those of their affiliated organizations, or those of the publisher, the editors and the reviewers. Any product that may be evaluated in this article, or claim that may be made by its manufacturer, is not guaranteed or endorsed by the publisher.

## References

[1] Thebaud B Goss KN Laughon M Whitsett JA Abman SH Steinhorn RH Bronchopulmonary dysplasia. *Nat Rev Dis Primers.* (2019) 5:78. 31727986 10.1038/s41572-019-0127-7 PMC6986462

[2] Jensen EA Schmidt B. Epidemiology of bronchopulmonary dysplasia. *Birth Defects Res A Clin Mol Teratol.* (2014) 100:145–57. 24639412 10.1002/bdra.23235 PMC8604158

[3] Van Marter LJ. Epidemiology of bronchopulmonary dysplasia. *Semin Fetal Neonatal Med.* (2009) 14:358–66. 19783238 10.1016/j.siny.2009.08.007

[4] Siffel C Kistler KD Lewis JFM Sarda SP. Global incidence of bronchopulmonary dysplasia among extremely preterm infants: a systematic literature review. *J Matern Fetal Neonatal Med.* (2021) 34:1721–31. 10.1080/14767058.2019.1646240 31397199

[5] Lewis T Chalise P Gauldin C Truog W. Pharmacometabolomics of respiratory phenotypic response to dexamethasone in preterm infants at risk for bronchopulmonary dysplasia. *Clin Transl Sci.* (2019) 12:591–9. 10.1111/cts.12659 31188532 PMC6853142

[6] Pang Z Chong J Zhou G de Lima Morais DA Chang L Barrette M MetaboAnalyst 5.0: narrowing the gap between raw spectra and functional insights. *Nucleic Acids Res.* (2021) 49:W388–96. 10.1093/nar/gkab382 34019663 PMC8265181

[7] Zhao YD Yin L Archer S Lu C Zhao G Yao Y Metabolic heterogeneity of idiopathic pulmonary fibrosis: a metabolomic study. *BMJ Open Respir Res.* (2017) 4:e000183. 10.1136/bmjresp-2017-000183 28883924 PMC5531310

[8] Gaugg MT Engler A Bregy L Nussbaumer-Ochsner Y Eiffert L Bruderer T Molecular breath analysis supports altered amino acid metabolism in idiopathic pulmonary fibrosis. *Respirology.* (2019) 24:437–44. 10.1111/resp.13465 30681243

[9] Liu X Wang J Dou P Zhang X Ran X Liu L The ameliorative effects of arctiin and arctigenin on the oxidative injury of lung induced by Silica via TLR-4/NLRP3/TGF-beta signaling pathway. *Oxid Med Cell Longev.* (2021) 2021:5598980. 10.1155/2021/5598980 34336106 PMC8313330

[10] Bloom B Stetten D Jr. The fraction of glucose catabolized via the glycolytic pathway. *J Biol Chem.* (1955) 212:555–63. 14353856

[11] Rohatgi N Nielsen TK Bjorn SP Axelsson I Paglia G Voldborg BG Biochemical characterization of human gluconokinase and the proposed metabolic impact of gluconic acid as determined by constraint based metabolic network analysis. *PLoS One.* (2014) 9:e98760. 10.1371/journal.pone.0098760 24896608 PMC4045858

[12] Plecita-Hlavata L D’Alessandro A El Kasmi K Li M Zhang H Jezek P Metabolic reprogramming and redox signaling in pulmonary hypertension. *Adv Exp Med Biol.* (2017) 967:241–60. 10.1007/978-3-319-63245-2_14 29047090

[13] D’Alessandro A El Kasmi KC Plecita-Hlavata L Jezek P Li M Zhang H Hallmarks of pulmonary hypertension: mesenchymal and inflammatory cell metabolic reprogramming. *Antioxid Redox Signal.* (2018) 28:230–50. 10.1089/ars.2017.7217 28637353 PMC5737722

[14] Gong J Feng Z Peterson AL Carr JF Lu X Zhao H The pentose phosphate pathway mediates hyperoxia-induced lung vascular dysgenesis and alveolar simplification in neonates. *JCI Insight.* (2021) 6:e137594. 10.1172/jci.insight.137594 33497360 PMC8021105

[15] Aboushousha R Elko E Chia SB Manuel AM van de Wetering C van der Velden J Glutathionylation chemistry promotes interleukin-1 beta-mediated glycolytic reprogramming and pro-inflammatory signaling in lung epithelial cells. *FASEB J.* (2021) 35:e21525. 10.1096/fj.202002687RR 33817836 PMC8073242

[16] Williams AF Jones M. Dexamethasone increases plasma amino acid concentrations in bronchopulmonary dysplasia. *Arch Dis Child.* (1992) 67:5–9. 10.1136/adc.67.1_spec_no.5 1536587 PMC1590343

[17] Tsai FJ Tsai CH Wu SF Liu YH Yeh TF. Catabolic effect in premature infants with early dexamethasone treatment. *Acta Paediatr.* (1996) 85:1487–90. 10.1111/j.1651-2227.1996.tb13957.x 9001663

[18] Montgomery AM Bazzy-Asaad A Asnes JD Bizzarro MJ Ehrenkranz RA Weismann CG. Biochemical screening for pulmonary hypertension in preterm infants with bronchopulmonary dysplasia. *Neonatology.* (2016) 109:190–4. 10.1159/000442043 26780635

[19] Trittmann JK Jin Y Chicoine LG Liu Y Chen B Nelin LD. An arginase-1 SNP that protects against the development of pulmonary hypertension in bronchopulmonary dysplasia enhances NO-mediated apoptosis in lymphocytes. *Physiol Rep.* (2016) 4:e13041. 10.14814/phy2.13041 27895230 PMC5358007

[20] Ronde E Reiss IKM Hankemeier T De Meij TG Frerichs N Schoenmakers S. The potential of metabolomic analyses as predictive biomarkers of preterm delivery: a systematic review. *Front Endocrinol (Lausanne).* (2021) 12:668417. 10.3389/fendo.2021.668417 34552554 PMC8451156

[21] Zhou Y Xu Y Zhang X Huang Q Tan W Yang Y Plasma levels of amino acids and derivatives in retinopathy of prematurity. *Int J Med Sci.* (2021) 18:3581–7. 10.7150/ijms.63603 34522185 PMC8436098

[22] Tarracchini C Milani C Longhi G Fontana F Mancabelli L Pintus R Unraveling the microbiome of necrotizing enterocolitis: insights in novel microbial and metabolomic biomarkers. *Microbiol Spectr.* (2021) 9:e0117621. 10.1128/Spectrum.01176-21 34704805 PMC8549755

[23] Wang L Zhong WH Liu DY Shen HQ He ZJ. Metabolic analysis of infants with bronchopulmonary dysplasia under early nutrition therapy: an observational cohort study. *Exp Biol Med (Maywood).* (2021) 1247:470–9. 10.1177/15353702211060513 34894806 PMC8943329

[24] Kontou A Virgiliou C Mouskeftara T Begou O Meikopoulos T Thomaidou A Plasma lipidomic and metabolomic profiling after birth in neonates born to SARS-CoV-19 infected and non-infected mothers at delivery: preliminary results. *Metabolites.* (2021) 11:830. 10.3390/metabo11120830 34940588 PMC8706054

